# GABAergic Neurons in the Rat Medial Septal Complex Express Relaxin-3 Receptor (RXFP3) mRNA

**DOI:** 10.3389/fnana.2017.00133

**Published:** 2018-01-17

**Authors:** Hector Albert-Gascó, Sherie Ma, Francisco Ros-Bernal, Ana M. Sánchez-Pérez, Andrew L. Gundlach, Francisco E. Olucha-Bordonau

**Affiliations:** ^1^Unitat Predepartamental de Medicina, Facultat de Ciències de la Salut, Universitat Jaume I, Castellón, Spain; ^2^The Florey Institute of Neuroscience and Mental Health, Parkville, VIC, Australia; ^3^Florey Department of Neuroscience and Mental Health, The University of Melbourne, Melbourne, VIC, Australia

**Keywords:** arousal, ChAT, emotion, GABA, hippocampus, nucleus incertus, relaxin-3, theta rhythm

## Abstract

The medial septum (MS) complex modulates hippocampal function and related behaviors. Septohippocampal projections promote and control different forms of hippocampal synchronization. Specifically, GABAergic and cholinergic projections targeting the hippocampal formation from the MS provide bursting discharges to promote theta rhythm, or tonic activity to promote gamma oscillations. In turn, the MS is targeted by ascending projections from the hypothalamus and brainstem. One of these projections arises from the nucleus incertus in the pontine tegmentum, which contains GABA neurons that co-express the neuropeptide relaxin-3 (Rln3). Both stimulation of the nucleus incertus and septal infusion of Rln3 receptor agonist peptides promotes hippocampal theta rhythm. The G_i/o_-protein-coupled receptor, relaxin-family peptide receptor 3 (RXFP3), is the cognate receptor for Rln3 and identification of the transmitter phenotype of neurons expressing RXFP3 in the septohippocampal system can provide further insights into the role of Rln3 transmission in the promotion of septohippocampal theta rhythm. Therefore, we used RNAscope multiplex *in situ* hybridization to characterize the septal neurons expressing *Rxfp3* mRNA in the rat. Our results demonstrate that *Rxfp3* mRNA is abundantly expressed in vesicular GABA transporter (*vGAT*) mRNA- and parvalbumin (*PV*) mRNA-positive GABA neurons in MS, whereas *ChAT* mRNA-positive acetylcholine neurons lack *Rxfp3* mRNA. Approximately 75% of *Rxfp3* mRNA-positive neurons expressed *vGAT* mRNA (and 22% were *PV* mRNA-positive), while the remaining 25% expressed *Rxfp3* mRNA only, consistent with a potential glutamatergic phenotype. Similar proportions were observed in the posterior septum. The occurrence of RXFP3 in PV-positive GABAergic neurons gives support to a role for the Rln3-RXFP3 system in septohippocampal theta rhythm.

## Introduction

Intrinsic neural circuits within, and projections from, the MS subserve various roles of this important brain area in different functions ranging from arousal, attention and spatial working memory (Givens and Olton, 1990; Sweeney et al., 1992; Osborne, 1994). Much research on the MS has centered on characterizing its projections to the hippocampus [see (Zaborszky et al., 2012, 2014) for review], in addition to descending projections from the MS to the hypothalamus, raphe nuclei and the NI (Borhegyi and Freund, 1998; Leranth et al., 1999; Sánchez-Pérez et al., 2015). Modulation of septal function has been traditionally viewed to derive strongly from ascending projections from the posterior hypothalamus and brainstem, including the raphe nuclei, which have been described as modulators of hippocampal theta rhythm via activation of the septohippocampal projection system (Vertes and Kocsis, 1997; Vertes, 2005). In addition, descending projections from the somatostatin-positive GABA projection neurons of the hippocampus provide a descending feedback regulation of the MS (Toth et al., 1993; Gulyas et al., 2003; Yuan et al., 2017).

However, the less well-studied projection from the NI in the pontine tegmentum also strongly modulates the MS (Goto et al., 2001; Olucha-Bordonau et al., 2003, 2012). Specifically, NI projections to the MS are associated with modulation of hippocampal theta rhythm. Electrical stimulation of the NI increased theta rhythm band power of the CA1 hippocampal field potential and NI lesions attenuated the increased hippocampal theta rhythm power induced by stimulation of the nucleus reticularis pontis oralis (RPO) in urethane-anesthetized rats (Nuñez et al., 2006).

A major population of GABA neurons in the NI co-express the neuropeptide, Rln3 (Ma et al., 2007) and NI projections and Rln3-positive fibers are in close contact with cholinergic and GABAergic neurons in the MS (Olucha-Bordonau et al., 2012). Moreover, infusion of a Rln3 analog into the MS increased hippocampal theta rhythm, whereas infusion of a Rln3 receptor antagonist impaired the theta rhythm produced by novel environment exploration or RPO stimulation (Ma et al., 2009). Different approaches in recent years have confirmed and extended these observations regarding the role of the NI and its associated peptide Rln3 in subcortical modulation of hippocampal theta rhythm, with an observed synchrony between the firing of NI neurons and different phases of hippocampal theta rhythm (Ma et al., 2013; Martínez-Bellver et al., 2015, 2017).

The cognate receptor for Rln3 is the G_i/o_-protein-coupled receptor, RXFP3. In *in vitro* studies in Chinese hamster ovary cells transfected with RXFP3, bath application of Rln3 results in inhibition of cAMP synthesis and increased ERK phosphorylation (Liu et al., 2003; van der Westhuizen et al., 2005, 2007; Bathgate et al., 2013). In agreement with a potential inhibitory effect of neuronal RXFP3 activation, Rln3 and a selective RXFP3 agonist, RXFP3-A2 (Shabanpoor et al., 2012), hyperpolarized RXFP3-expressing magnocellular neurons in the rat paraventricular and supraoptic hypothalamic nuclei (Kania et al., 2017). Furthermore, following intracerebroventricular (icv) infusion of RXFP3-A2, we observed increased phospho-ERK levels in the MS and disruption of spatial working memory in a spatial alternation test (Albert-Gascó et al., 2017), although the precise relationship between these effects is not known.

The MS is composed of a heterogeneous population of neurons and each neuronal type participates in a different way in septo-hippocampal interactions (Sotty et al., 2003). For example, slow firing cholinergic neurons facilitate hippocampal activity (Sotty et al., 2003), while PV GABAergic projection neurons inhibit hippocampal interneurons (Toth et al., 1997). Somatostatin positive neurons are concentrated in the HDB (Köhler and Eriksson, 1984), but to our knowledge, no functional role has been assigned to these neurons. Different types of calcium-binding protein-expressing neurons and neurons expressing choline acetyltransferase (ChAT) are targeted by NI axons/terminals in the rat (Olucha-Bordonau et al., 2012), but it is not clear which of these neurons express RXFP3. Thus, we explored the distribution of *Rxfp3* mRNA expression in different neuronal types of the rat septal area using multiplex *in situ* hybridization and specific probes for *Rxfp3, ChAT, vGAT* (*slc32a1*), *PV*, and *somatostatin (SOM)* transcripts.

## Materials and Methods

### Animals

Experiments were conducted with approval from The Florey Institute of Neuroscience and Mental Health Animal Ethics Committee, in compliance with guidelines of the National Health and Medical Research Council of Australia. Adult male Sprague-Dawley rats weighing 300–320 g were maintained on a 12–12 h light-dark cycle with lights on at 0700 h. Rats were provided free access to food and water.

### Multiplex *in Situ* Hybridization (ISH)

The distribution of septal *Rxfp3* mRNA-positive neurons and their GABAergic or cholinergic phenotype was assessed using RNAscope multiplex *in situ* hybridization. RNAscope^®^ is a commercial method provided by Advanced Cell Diagnostics (ACD, Newark, CA, United States), which involves the incubation of post-fixed, fresh-frozen brain sections with up to three custom probes. Standard probes contain 20 ZZ pairs (25 base pairs/Z) which cover a total of ∼1000 base pairs of the target mRNA. *In silico* verification of the probes is performed and validated to select oligonucleotides with compatible melting temperature for optimal hybridization under RNAscope assay conditions and minimal cross-hybridization to off-target sequences. There is a verification procedure conducted following each major step during the probe design to guarantee accuracy, according to previously described rules (Wang et al., 2012).

Two naïve rats were deeply anesthetized with pentobarbitone (100 mg/kg, i.p.), decapitated, and brains were quickly extracted and rapidly frozen on dry ice. The fresh-frozen brains were embedded in OCT embedding gel (Tissue-Tek^®^ OCT, Optimum Cutting Temperature, Sakura Finetek USA, Inc., Torrance, CA, United States) and stored at -80°C. Before cryo-sectioning, brains were warmed to -20°C for 2 h and then mounted on a cryostat (Cryocut CM 1800, Leica Microsystems, North Ryde, NSW, Australia) using OCT embedding gel. Coronal sections (16 μm) were cut and thaw-mounted on Superfrost-Plus Slides (Fisher Scientific, Hampton, NH, United States, Cat#12-550-15).

Sections were fixed in 4% paraformaldehyde (PFA) for 16 min at 4°C, rinsed in PBS, and dehydrated in increasing ethanol concentrations (50, 70, and 100%). Once dehydrated the sections were stored in 100% ethanol overnight at -20°C. The next day, slides were air-dried and a hydrophobic barrier was drawn around the sections (ImmEdge hydrophobic PAP pen, Vector Laboratories, Burlingame, CA, United States; Cat #310018). Sections were incubated with protease pretreatment-4 (ACD, Cat #322340) for 16 min. After a PBS rinse, sections were incubated for 2 h at 40°C with three different probe combinations targeting (i) *Rxfp3* (ACD, #316181), *ChAT* (ACD, #430111), and *vGAT* (*Slc32a1*; ACD, #424541) mRNA; (ii) *Rxfp3, PV* (*pvalb*, ACD, #407828) and *SOM* (Sst, ACD, #412181-C3) mRNA; (iii) *Rxfp3, PV*, and *vGAT* mRNA. Sections were processed in two different trials. Following incubation, sections were rinsed with wash buffer (ACD, Cat#310091) and signals were amplified with ACD amplifier reagents according to manufacturer’s protocol. After 2 × rinses with wash buffer, sections were stained with DAPI (ACD, #320851), covered with fluorescent mounting medium (Fluoromount-G, Southern Biotech, Birmingham, AL, United States, Cat# 17985-10), coverslipped, and stored at -20°C.

### Imaging and Quantification of Co-expression of Transcripts

Fluorescence images were taken with an LSM 780 Zeiss Axio Imager 2 confocal laser scanning microscope (Carl Zeiss AG, Jena, Germany). The system is equipped with a stitching stage, and Zen software (Carl Zeiss AG) was used to stitch tiled images taken with a 20 × objective. Quantification of cellular colocalization of transcripts (one section/bregma level, rat and probe combination) was conducted manually using Fiji (Schindelin et al., 2012) [Note: Results consistent with those observed in the sections assessed, were also observed in adjacent brain areas, and in other rat brains, for all probes]. The total number of positive neurons for each region was counted separately, relative to DAPI-stained nuclei, to avoid bias. The percentage co-expression of transcripts was related to the total number of *Rxfp3* mRNA-positive neurons in each of the septal areas. Higher-power, (inset) images to illustrate co-localization were taken using a 40 × objective.

## Results

In these experiments, we assessed the rostrocaudal distribution of *Rxfp3* mRNA-positive neurons in the MS/diagonal band, LS, triangular septal nucleus, and SFi, and determined whether these *Rxfp3* mRNA-positive neurons co-expressed *ChAT* or *vGAT* mRNA or *PV* and/or *vGAT* mRNA (or *SOM* and/or *vGAT* mRNA). All these neurotransmitter-related transcripts and their related proteins or peptides have been described as clear markers of the onion-like structure of the septum. According to Wei et al. (2012), the onion-like MS can be described as a five-layer structure with layers determined by their highest density marker (MS-1-MS-3, LSv, and LSi). Layers are distributed from the midline to the LSi with MS-1 on the midline, rich in PV neurons; followed by MS-2, rich in ChAT neurons; followed by MS-3, rich in nNOS; followed by CR (LSV), and CB (LSi). The following results illustrate a high level of co-localization of *Rxfp3* and *vGAT* mRNA in neurons in most septal regions. In contrast, in caudal septal regions and diagonal band, no co-localization of *Rxfp3* with *vGAT* mRNA occurred, suggesting an alternative non-GABAergic phenotype (**Table 1**).

**Table 1:** Semi-quantification of the number of neurons expressing *Rxfp3, vGAT*, and *ChAT* mRNA alone and in combination throughout the different regions of the rat septal area.

Bregma	Area	*Rxfp3*	*vGAT*	*PV*	*SOM*	*ChAT*	*Rxfp3/vGAT*	*Rxfp3/PV/vGAT*	*Rxfp3/ChAT*	*Rxfp3/-*
1.08 mm	MS	77 ± 28	240	–	–	91	44	–	0	5
			–	116	0	–	–	39	–	66
	VDB	67 ± 14	148	–	–	41	45	–	3	5
			–	106	0	–	–	37	–	35
	HDB	62	–	16	78	–	–	1	–	53
0.6 mm	MS	107 ± 13	500 ± 100	–	–	75	82	7	0	14
				179	–	–	99	–	–	12
	VDB	54 ± 12	89	–	–	9	31	–	0	34
			237	87	–	–	23	4	–	15
	HDB	37 ± 6	162	–	–	26	21	–	0	10
			234	43	–	–	24	7	–	12
0.48 mm	MS	172 ± 16	1500 ± 30	126 ± 10	–	–	109 ± 8	27 ± 8	–	22 ± 15
	VDB	50	306 ± 20	83 ± 15	–	–	24	14	–	12
	HDB	41.5 ± 13	188 ± 16	23 ± 5	–	–	23 ± 10	7 ± 2	–	12 ± 5
0.24 mm	LSI	11	37	–	–	2	9	–	0	2
	SFi	20	97	–	–	2	19	–	0	1
	MS	8	86	–	–	4	8	–	0	0
-0.24 mm	LSD	14	61	–	–	0	12	–	0	2
	SFi	100	314	–	–	0	75	–	0	25
	TS	245	800	–	–	3	193	–	0	52
	LSV	223	307	–	–	4	142	–	1	80
	SFO	30	4	–		6	2	–	0	28

*– Mean ± SEM are indicated on cases where more than one subject was analyzed. – indicates not determined probe combinations and split rows indicate each of different trials.*

### *Rxfp3* mRNA-Positive Neurons in MS Co-express *vGAT*, But Not *ChAT* mRNA

**FIGURE 1:**
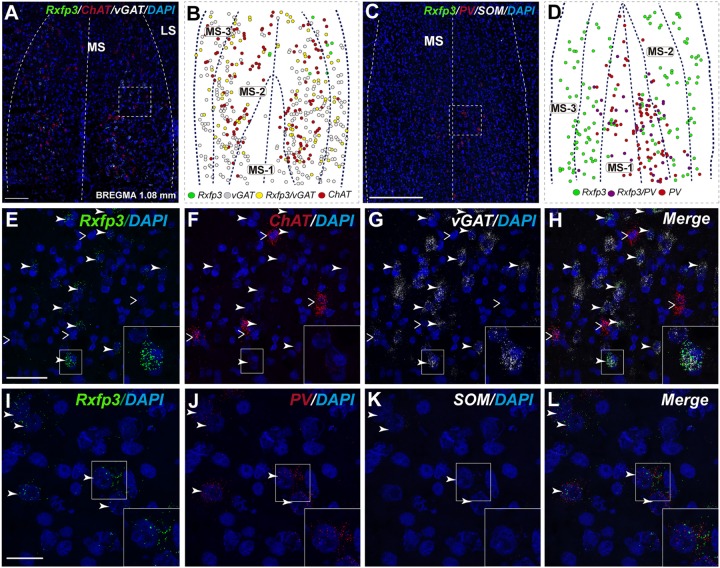
Distribution of neurons expressing *Rxfp3, vGAT* (*slc32a1*), and *ChAT* mRNA relative to DAPI-stained nuclei, and *Rxfp3, SOM*, and *PV* mRNA in the rat MS at bregma +1.08 mm **(A,C)**; and a schematic map illustrating the different neuronal phenotypes based on mRNA co-expression, and their distribution **(B,D)**. Thick dotted lines indicate the midline and the medial and lateral septal border and thin dotted lines, the layers within MS. Higher magnification images illustrating co-localization of *Rxfp3* **(E)**, *ChAT* **(F)**, and *vGAT* **(G)** mRNA and merged signals **(H)**. High-magnification images illustrating the co-localization of *Rxfp3* **(I)**, *PV* **(J)**, and *SOM* **(K)** mRNA and merged signals **(L)**. Arrowheads indicate neurons double-labeled for *Rxfp3* and *vGAT* mRNA **(E,F)** and *Rxfp3* and *PV* mRNA **(I,J)**. No colocalization of *Rxfp3* and *ChAT* mRNA was observed (open arrowheads). Insets (lower right) are high magnification images of the boxed area in **(E–L)**, illustrating a neuron double-labeled for *Rxfp3* and *vGAT* mRNA or *Rxfp3* and *PV* mRNA consistent with a GABAergic phenotype. Calibration bar in **(A)** 250 μm, **(C)** 250 μm, **(E–H)** 50 μm, and **(I–L)** 20 μm.

**FIGURE 2:**
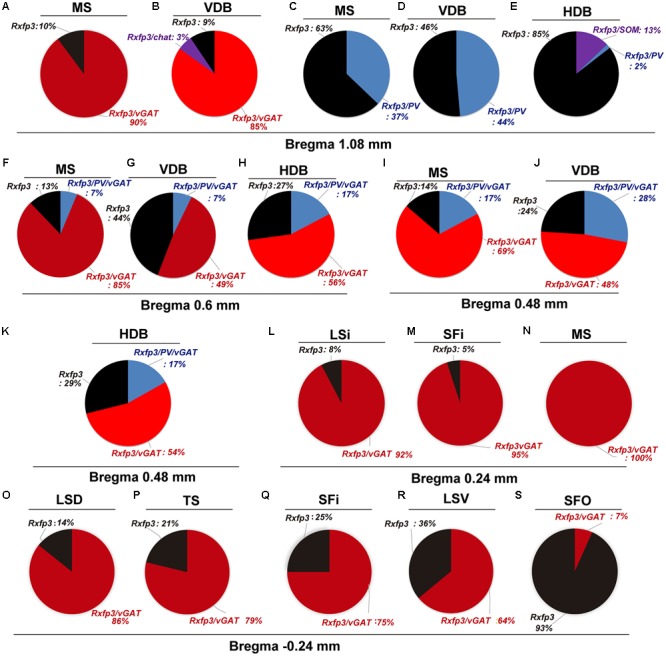
Percentage co-localization of *Rxfp3, vGAT, PV*, and *SOM* mRNA throughout the septal complex. Rostral to caudal levels of MS **(A,C)**, VDB **(B,D)**, and HDB **(E)** at +1.08 mm from bregma; MS **(F)**, VDB **(G)** and HDB **(H)** at +0.6 mm from bregma; MS **(I)**, VDB **(J)** and HDB **(K)** at +0.48 mm from bregma; LSi **(L)**, SFi **(M)** and MS **(N)** at +0.24 mm from bregma; and LSD **(O)**, TS **(P)**, SFi **(Q)**, LSV **(R)** and SFO **(S)** at –0.24 mm from bregma.

At the most rostral level of the MS (bregma ∼1.08 mm), *Rxfp3* mRNA-expressing neurons were mainly located between the MS-1 and MS-3 layers (**Figures 1A–D**). The majority of *Rxfp3* mRNA-positive neurons in these layers co-expressed *vGAT* mRNA (∼90%; 44/49) of expressing neurons) while only ∼10% (5/49 neurons) of *Rxfp3* mRNA-positive neurons lacked *vGAT* and *ChAT* mRNA (**Figures 1E–H, 2**). Given the distribution of these neurons and the co-localization of *Rxfp3* mRNA with *vGAT* and not *ChAT* mRNA, this labeling is consistent with expression of RXFP3 by GABA neurons (Ma et al., 2009, 2017; Olucha-Bordonau et al., 2012). With a different combination of probes for *Rxfp3*/*PV*/*SOM* mRNA, ∼37% of *Rxfp3* mRNA-expressing-neurons in the MS (39/105 neurons), expressed *PV* mRNA, and were distributed within MS-1, while 63% (66/105) of *Rxfp3* mRNA-positive neurons that did not co-express *PV* mRNA (**Figures 1I–L**), were located within MS-2 and 3 (**Figure 1D**).

**FIGURE 3:**
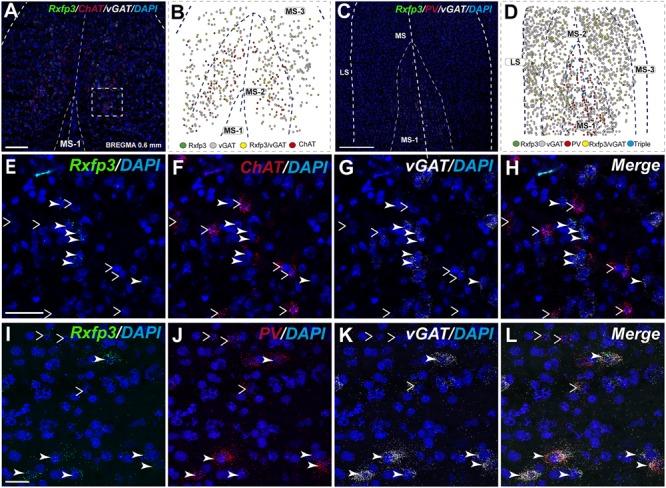
Distribution of neurons expressing *Rxfp3, vGAT* (*slc32a1*), *ChAT*, and *PV* mRNA relative to DAPI-stained nuclei in the rat MS at bregma +0.60 mm **(A,C)**, and a schematic map illustrating the different neuronal phenotypes based on mRNA co-expression, and their distribution **(B,D)**. Thick dotted lines indicate the midline and the medial and lateral septal border and thin dotted lines, the layers within MS. High-magnification images illustrating colocalization of *Rxfp3* **(E)**, *ChAT* **(F)**, and *vGAT* **(G)** mRNA, and merged signals **(H)**. High-magnification images illustrating co-localization of *Rxfp3* **(I)**, *PV* **(J)**, and *vGAT* **(K)** mRNA and merged signals **(L)**. Arrowheads indicate neurons double-labeled for *Rxfp3* and *vGAT* mRNA **(E–H)** and *Rxfp3, vGAT and PV* mRNA **(I–L)**. No colocalization of *Rxfp3* and *ChAT* mRNA was observed (open arrowheads). Calibration bar in **(A)** 250 μm, **(C)** 250 μm, **(E–H)** 50 μm, and **(I–L)**.

In the mid-anterior dorsal part of the MS (bregma ∼0.6 mm), *Rxfp3* mRNA-expressing neurons were present mainly in the MS-1 layer, characterized as containing PV neurons, and in more lateral layers containing lower PV neuron densities (Kiss et al., 1990; Wei et al., 2012). The highest number of *Rxfp3* mRNA-expressing neurons was located between MS-2 and MS-3. In the ventral part of this mid-MS level, *Rxfp3* mRNA-positive neurons were limited to the MS-2 (**Figures 3A–D**). At this level, the majority of *Rxfp3* mRNA-expressing neurons co-expressed *vGAT* mRNA (∼87% (82/94) of labeled neurons), while only ∼13% (13/94 neurons) of labeled cells expressed *Rxfp3* mRNA in the absence of *vGAT and ChAT* mRNA (**Figures 3E–H**). In sections labeled with the *Rxfp3*/*PV*/*vGAT* probe combination, some *Rxfp3* mRNA-expressing-neurons co-expressed *PV* mRNA (∼6%; 7/111 neurons), but most co-expressed *vGAT* mRNA, distributed within MS-2 (82% (91/111) of labeled neurons). Only 12% (14/111 neurons) of *Rxfp3* mRNA-expressing neurons did not co-express either transcript (**Figures 2, 3I–L**).

**FIGURE 4:**
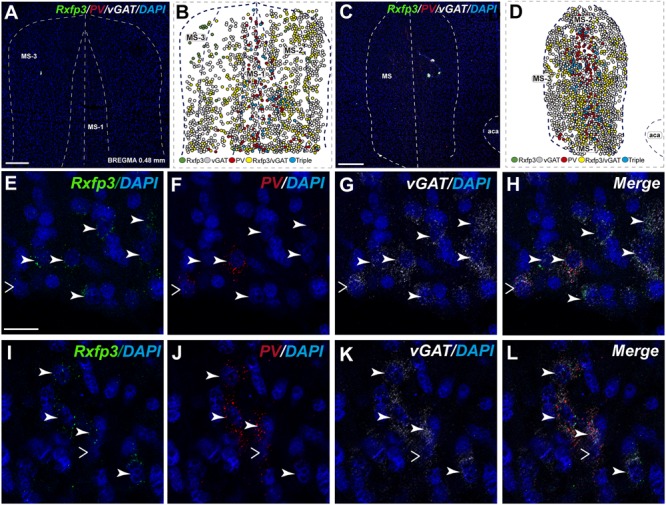
Distribution of neurons expressing *Rxfp3, vGAT* (*slc32a1*), *ChAT*, and *PV* mRNA relative to DAPI-stained nuclei in the rat MS at bregma +0.48 mm **(A,C)**, and a schematic map illustrating the different neuronal phenotypes based on mRNA co-expression, and their distribution **(B,D)**. Thick dotted lines indicate the midline and the medial and lateral septal border and thin dotted lines the layers within MS. High-magnification images illustrating colocalization of *Rxfp3* **(E,I)**, *PV* **(F,J)**, and *vGAT* **(G,K)** mRNA and merged signals **(H,L)**. Arrowheads indicate neurons double-labeled for *Rxfp3* and *vGAT* mRNA **(E–H)** and *Rxfp3, vGAT, and PV* mRNA **(I–L)**. No colocalization of *Rxfp3* and *PV* mRNA was indicated with open arrowheads. Calibration bar in **(A)** 200, **(C)** 500, and **(E–L)** 50 μm.

In the mid-posterior part of the septal area (bregma ∼0.48 mm), the *Rxfp3*/*PV*/*SOM* probe combination revealed that *Rxfp3* mRNA-expressing neurons were distributed across MS-1 to MS-3. *Rxfp3* mRNA-positive neurons that co-expressed *PV* mRNA (17%; 27/157 neurons) were mostly located near the midline (**Figures 4A–D**). In the MS-2 and MS-3 layers, 69% (109/157 neurons) of *Rxfp3* mRNA-positive neurons co-expressed *vGAT* mRNA and 14% (22/172 neurons) did not co-express either transcript (**Figures 2**,‘**4E–L**).

**FIGURE 5:**
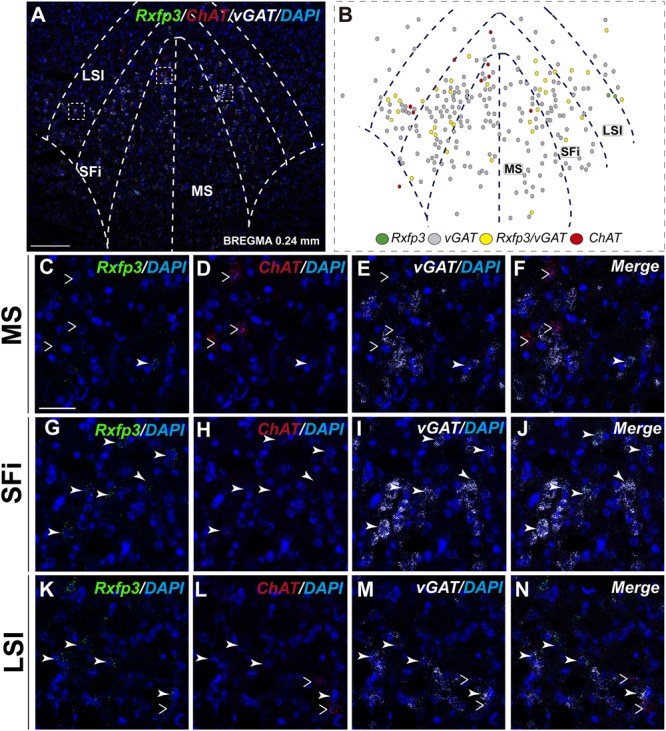
Distribution of neurons expressing *Rxfp3, vGAT* (*slc32a1*), and *ChAT* mRNA, relative to DAPI-stained nuclei in the rat MS, SFi, and LSI at bregma +0.24 mm **(A)** and a schematic map illustrating different neuronal phenotypes based on mRNA co-expression, and their distribution **(B)**. Dotted lines indicate the midline and the medial and lateral septal and septofimbrial borders. High-magnification images illustrate co-localization of *Rxfp3* **(C,G,K)**, *ChAT* **(D,H,L)**, *vGAT* **(E,I,M)** mRNA and merged signals **(F,J,N)** in the MS, SFi, and LSI, respectively. No co-localization of *Rxfp3* and *ChAT* mRNA was observed (open arrowheads). Calibration bar in **(A)** 125 μm and **(C–N)** 50 μm.

In the posterior septum (bregma ∼0.24 mm), *Rxfp3* mRNA-positive neurons were present in the MS and were more dense in the SFi and the LSI (**Figures 5A,B**). In the MS, *Rxfp3* mRNA-positive neurons co-expressed *vGAT* mRNA in ∼100% of cases (8/8 neurons), but did not express *ChAT* mRNA (**Figures 2, 5C–F**). In the SFi ∼95% (19/20 neurons) of *Rxfp3* mRNA-positive neurons co-expressed *vGAT* mRNA, and ∼5% (1/20 neurons) of *Rxfp3* mRNA-positive neurons lacked *vGAT* and *ChAT* mRNAs (**Figures 2, 5G–J**). Finally, in the LSI ∼92% (24/26 neurons) of detected neurons co-expressed *Rxfp3* and *vGAT* mRNAs whereas only ∼8% (2/26 neurons) of *Rxfp3* mRNA-positive neurons lacked *vGAT* and *ChAT* mRNA (**Figures 2, 5K–N**).

### Diagonal Band Neurons Co-express *Rxfp3* and *vGAT (slc32a1)* mRNA

**FIGURE 6:**
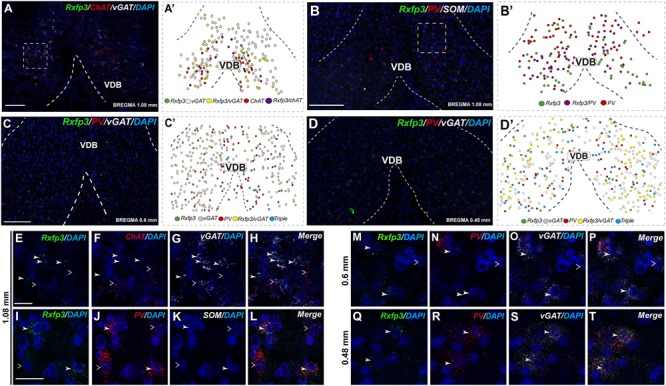
Distribution of neurons expressing *Rxfp3, vGAT* (*slc32a1*), *ChAT*, and *PV* mRNAs relative to DAPI-stained nuclei in the vertical limb of the diagonal band at bregma +1.08 **(A,B)**, +0.6 **(C)**, and 0.48 mm **(D)** and schematic map illustrating different colocalization neuronal phenotypes and their distribution **(A′–D′)**. Dotted lines indicate the midline and the VDB border. Higher magnification images illustrate colocalization of *Rxfp3* **(E)**, *ChAT* **(F)**, *vGAT* mRNA **(G)**, and merged signals **(H)**. High-magnification images illustrate co-localization of *Rxfp3* **(I,M,Q)**, *PV* **(J,N,R)**, SOM **(K)** *vGAT* **(O,S)** mRNA and merged signals **(L,P,T)** at different bregma levels. Arrowheads indicate neurons double-labeled for *Rxfp3* and *vGAT* **(E–H)** or *Rxfp3, vGAT*, and *PV* **(I–T)** mRNA and open arrowheads indicate *ChAT* or *PV* mRNA-positive neurons that do not express *Rxfp3* mRNA. Calibration bar in **(A)** 100 μm, **(B–D)** 125 μm, **(E–H)** 50 μm, and **(I–T)** 20 μm.

**FIGURE 7:**
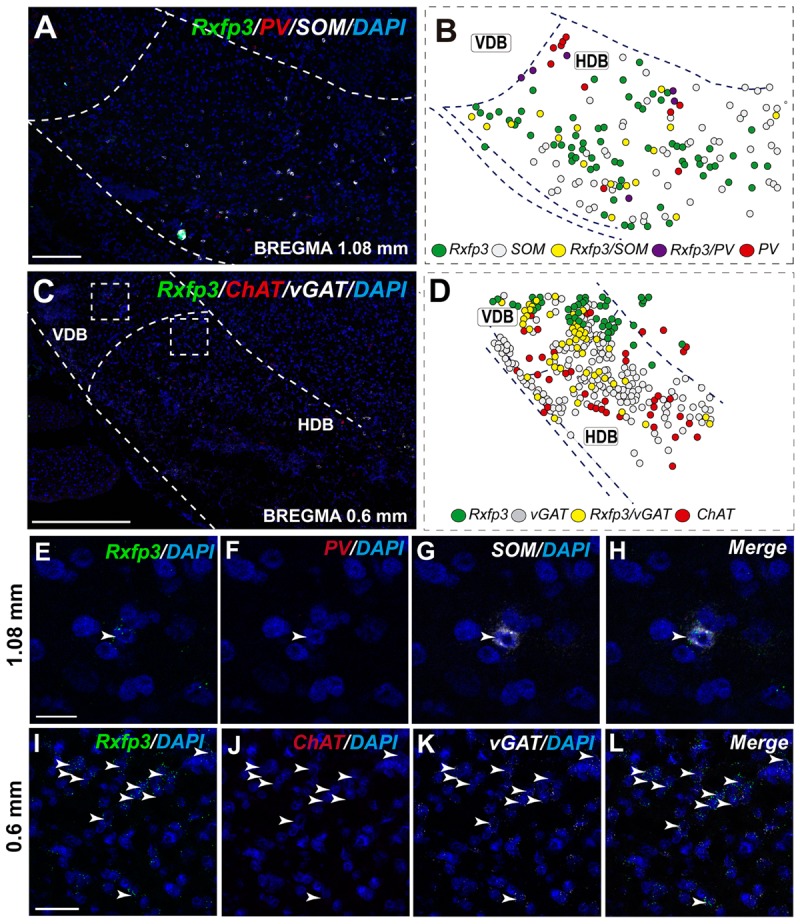
Distribution of neurons expressing *Rxfp3, vGAT* (*slc32a1*), *ChAT*, and *PV* mRNAs relative to DAPI-stained nuclei in the HDB at bregma +1.08 mm **(A)** +0.60 mm **(C)**, and schematic map illustrating different colocalization neuronal phenotypes and their distribution **(B,D)**. Dotted lines indicate the vertical and horizontal limbs of the diagonal band border. Higher magnification images illustrate colocalization of *Rxfp3* **(E)**, *PV* **(F)**, *SOM* mRNA **(G)**, and merged signals **(H)**; *Rxfp3* **(I)**, *ChAT* **(J)**, *vGAT* mRNA **(K)**, and merged signals **(L)** in the HDB, respectively. Arrowheads indicate neurons double-labeled for *Rxfp3, SOM*, and *PV* **(E–H)** or *Rxfp3* and *vGAT* **(I–L)**. No colocalization of *Rxfp3* and *ChAT* mRNAs was observed. Calibration bar in **(A)** 125 μm, **(C)** 200 μm, **(E–H)** 20 μm, and **(I–L)** 50 μm.

In the anterior (VDB; bregma ∼1.08 mm), *Rxfp3* mRNA-positive neurons were evenly distributed laterally at a similar distance from the midline (**Figures 6A–D′**). *Rxfp3* mRNA-positive neurons were present in the vicinity of *ChAT* mRNA-expressing neurons, but *Rxfp3* and *ChAT* mRNA were sparsely co-expressed in ∼6% (3/53 neurons) of total *Rxfp3* mRNA-positive cells, while ∼85% (45/53 neurons) co-expressed *Rxfp3* and *vGAT* mRNA, and *Rxfp3* transcripts were present in the absence of the other markers in only ∼9% (5/53 neurons) of identified neurons (**Figures 2, 6E–H**). In sections incubated in a different combination of probes, *Rxfp3* mRNA-expressing neurons in the VDB co-localized with *PV* mRNA (44%; 35/80 neurons) and did not co-localize with any marker in 46% (37/80) of neurons (**Figures 2, 6I–L**) In contrast, in the HDB, some *Rxfp3* mRNA-expressing neurons co-expressed *SOM* (13%; 8/62) and *PV* (2% 1/62) mRNA, but most did not co-express either of these transcripts (85%; 53/62 neurons) (**Figures 2, 7A,B,E–H**).

At more posterior levels (bregma ∼0.6 mm), *Rxfp3* mRNA-positive neurons were present in the VDB and the HDB (**Figures 6C,C′, 7C,D**). In the VDB, *Rxfp3* mRNA-expressing neurons were present in two clusters. From the total amount of Rxfp3 mRNA positive neurons ∼48% (31/65) of them co-expressed *vGAT* mRNA. A second cluster/population of *Rxfp3* mRNA-positive neurons, ∼52%; 34/65 neurons, did not co-express *vGAT* or *ChAT* mRNA. In sections labeled for *Rxfp3*/*PV*/*vGAT* mRNA some *Rxfp3* mRNA-expressing neurons expressed *PV* mRNA (7%; 4/56), while 49% (27/56) expressed *vGAT* mRNA and 44% (25/56) did not express either of the other transcripts (**Figures 2, 6M–P**).

**FIGURE 8:**
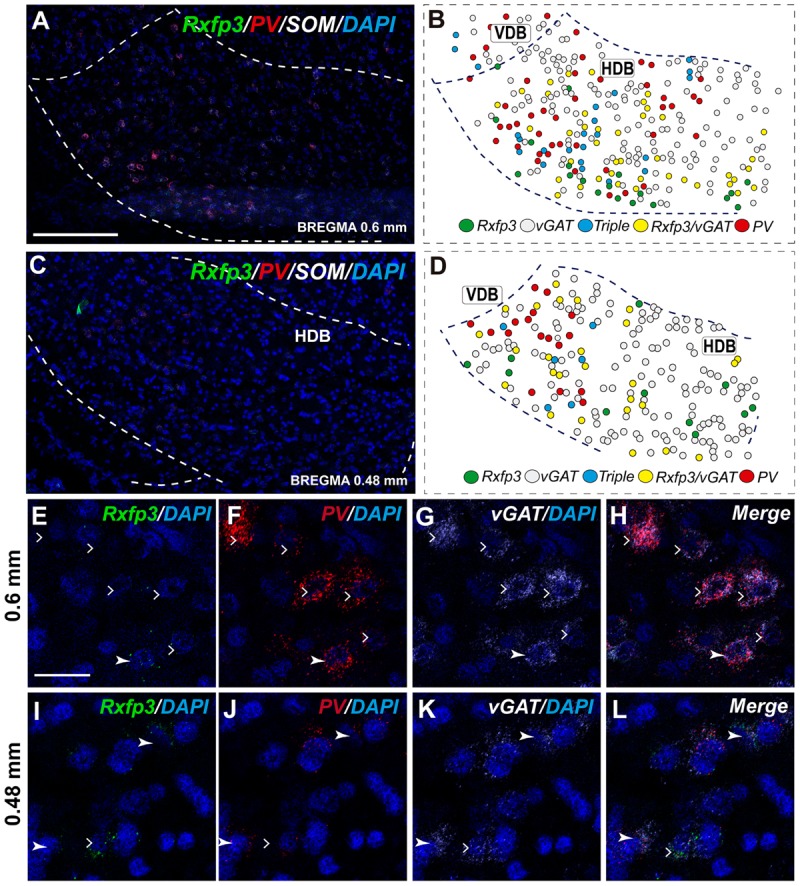
Distribution of neurons expressing *Rxfp3, vGAT* (*slc32a1*), *ChAT*, and *PV* mRNAs relative to DAPI-stained nuclei in the HDB at bregma +0.6 mm **(A)** +0.48 mm **(C)** and schematic map illustrating different colocalization neuronal phenotypes and their distribution **(B,D)**. Dotted lines indicate the vertical and horizontal limbs of the diagonal band border. Higher magnification images illustrate colocalization of *Rxfp3* **(E)**, *PV* **(F)**, *vGAT* mRNA **(G)**, and merged signals **(H)**; *Rxfp3* **(I)**, *PV* **(J)**, *vGAT* mRNA **(K)**, and merged signals **(L)** in the HDB, respectively. Arrowheads indicate neurons double-labeled for *Rxfp3, vGAT*, and *PV* **(E–H)** or *Rxfp3* and *vGAT* **(I–L)**. Lack of colocalization of *Rxfp3* and *PV* mRNA indicated with open arrowheads. Calibration bar in **(A,C)** 125 μm, **(E–L)** 20 μm.

The number of *Rxfp3* mRNA-positive neurons in the HDB was lower than in the VDB (**Figures 7C,D**). In contrast to the VDB, in the HDB the majority (∼68%; 21/31 neurons) of *Rxfp3* mRNA-positive neurons expressed *vGAT* mRNA, while the remaining were *vGAT* mRNA and *ChAT* mRNA negative (∼32%; 10/31 neurons) (**Figures 2, 7I–L**). *Rxfp3* mRNA colocalized with *vGAT* mRNA (56%; 23/41 neurons), and with *PV*/*vGAT* mRNA (17%; 7/41 neurons) and was also expressed in the absence of either transcript (27%; 11/41) (**Figures 2, 8A,B,E–H**).

In the mid-posterior part of the septal area (bregma ∼0.48 mm) sections labeled with *Rxfp3*/*PV*/*vGAT* probes displayed *Rxfp3* mRNA expressing neurons in the VDB (**Figures 6D,D′**) that co-expressed *PV*/*vGAT* mRNA (28%; 14/50 neurons), and *vGAT* mRNA (48%; 24/50 neurons) (**Figures 2, 6Q–T**), but some *Rxfp3* mRNA-positive neurons did not express either transcript (24%; 12/50 neurons). Likewise, analysis of the HDB, revealed that the majority of *Rxfp3* mRNA-positive neurons expressed *vGAT* mRNA (54%; 23/42 neurons) and a small proportion expressed *PV*/*vGAT* mRNA (17%; 7/42 neurons) or neither of the other transcripts (29%; 12/42 neurons) (**Figures 2, 8I–L**).

### Triangular Septal Area, and Septofimbrial and Dorsolateral Septal Area Contain Heterogeneous Populations of *Rxfp3* mRNA-Positive Neurons

**FIGURE 9:**
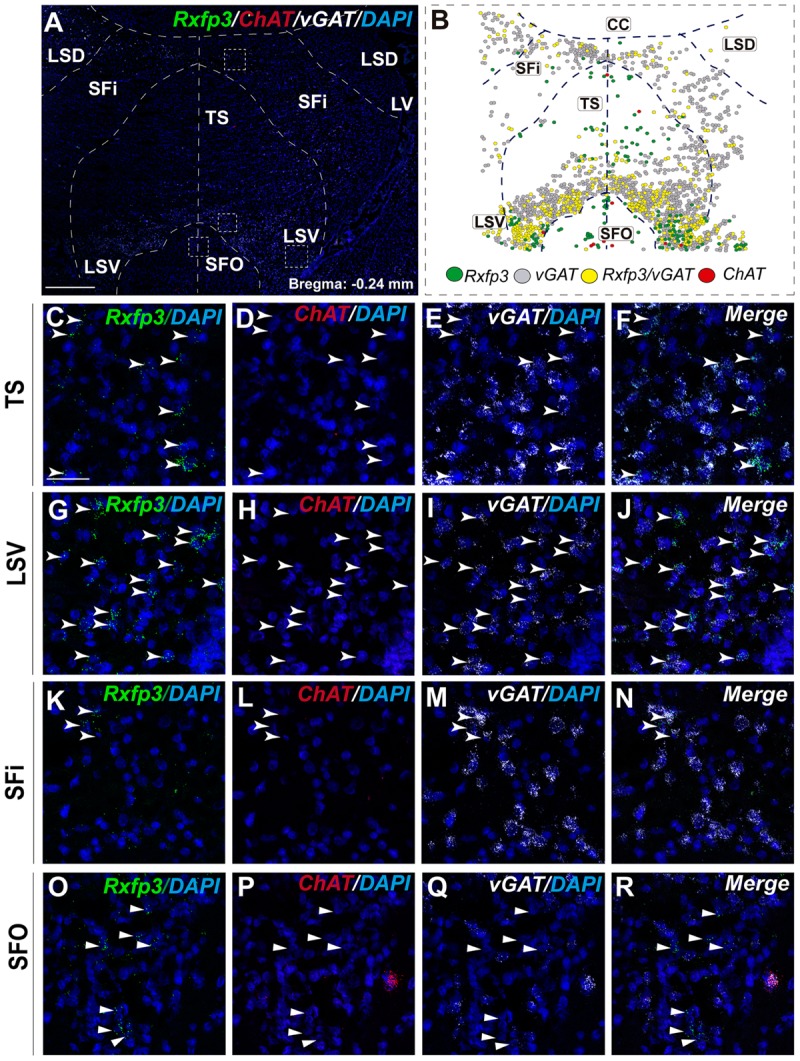
Distribution of neurons expressing *Rxfp3, vGAT* (*slc32a1*), and *ChAT* mRNA, relative to DAPI-stained nuclei in LSD, SFi, TS, LSV, and SFO at bregma –0.24 mm **(A)**, and a schematic map illustrating the different neuronal phenotypes and their distribution **(B)**. Dotted lines indicate the midline and borders between the different regions. High-magnification images illustrate the co-localization of *Rxfp3* **(C,G,K,O)**, *ChAT* **(D,H,L,P)**, *vGAT* **(E,I,M,Q)** mRNA and merged signals **(F,J,N,R)** in the TS, LSV, SFi, and SFO, respectively. Arrowheads indicate neurons double-labeled for *Rxfp3* and *vGAT* mRNA. No co-localization of *Rxfp3* and *ChAT* mRNA was observed (open arrowheads). Calibration bar in **(A)** 500 μm and **(C–R)** 50 μm.

In the most caudal region of the septum analyzed (bregma -0.24 mm), the distribution and phenotype of *Rxfp3* mRNA-positive neurons varied within the different nuclei. In the LSD, *Rxfp3* mRNA-positive neurons were widely and evenly distributed (**Figures 9A,B**) and were mainly *vGAT* mRNA-positive (∼86%; 12/14 neurons), with a small number of neurons located near the SFi that were *vGAT* mRNA negative (∼14%; 2/14 neurons; **Figures 2, 9K–N**).

*Rxfp3* mRNA-positive neurons in the SFi were mainly distributed in the most dorsal part of the nucleus near the corpus callosum (cc). In the ventral SFi, *Rxfp3* mRNA-positive neurons were lower in number (**Figures 9A,B**). Throughout the dorsal and ventral SFi, the majority of *Rxfp3* mRNA-positive neurons co-expressed *vGAT* mRNA (∼75%; 75/100 neurons; **Figures 2, 9L–O**).

The triangular septal area (TS) contained three *Rxfp3* mRNA-positive neuron populations based on their differential phenotype and distribution. Dispersed *Rxfp3* mRNA-positive neurons were present in the most dorsal portion near the midline, while in the most ventral TS, a large, densely packed population of *Rxfp3* mRNA-positive neurons were distributed alongside the border with the SFO (**Figures 9A,B**). The ventral TS area was rich in *vGAT* mRNA-expressing neurons, while the dorsal TS was not. *Rxfp3* mRNA-positive neurons in the dorsal TS were generally *vGAT* mRNA-negative, while in the ventral TS, *Rxfp3* mRNA-positive neurons were generally *vGAT* mRNA-positive. In the lateral part of the ventral TS, there was a population of *Rxfp3* mRNA-positive neurons which were *vGAT* mRNA-negative. Overall, ∼79% of *Rxfp3* mRNA-positive neurons in TS co-expressed *vGAT* mRNA (193/245 neurons), while the remainder were negative (∼21%; 52/245 neurons; **Figures 2, 9C–K**).

Similar to ventral TS, the LSV contained a large population of *vGAT* mRNA-positive neurons and most were *Rxfp3*/*vGAT* mRNA-positive (64%; 142/223 neurons). In addition to these GABAergic neurons, this area also contained a large non-GABAergic population (36%; 80/223 neurons; **Figures 2, 9G,H**). Finally, we noted that within the SFO, a vast majority of *Rxfp3* mRNA-positive neurons were *vGAT* mRNA-negative (93%; 28/30 cells; **Figures 2, 9O–R**).

## Discussion

In this study we have employed RNAscope multiplex *in situ* hybridization (Wang et al., 2014a,b; Li and Kim, 2015) to characterize the neurochemical phenotype of *Rxfp3* mRNA-positive neurons in the rat septal area. The highly specific nature of the method means that these data represent a more accurate estimation of the distribution of RXFP3 than studies using putative antisera against the receptor protein (Meadows and Byrnes, 2014), although this powerful approach does not provide information about the subcellular location of RXFP3, which might be available with alternative protein detection methods.

In the septal area, neurons expressing *Rxfp3* transcripts were concentrated in the MS complex, including the diagonal band nuclei, and in the posterior septum, including the SFi and TS nuclei. Some *Rxfp3* mRNA-positive neurons were also detected in LS divisions. Our findings are consistent with previous studies of the presence and distribution of *Rxfp3* mRNA in the rat septal area detected using radioactive oligonucleotide probes. Specifically, MS and HDB displayed moderate to high levels of *Rxfp3* mRNA, while in LSI and VDB, expression was moderate (Sutton et al., 2004; Ma et al., 2007). These findings are consistent with concurrent studies of these and other transcripts in rat hippocampus (Ma and Gundlach, unpublished data). Therefore, in light of the strong innervation of the rat septal region by Rln3-positive nerve fibers, the presence of septal RXFP3 binding sites, and functional studies (Ma et al., 2007, 2009), we conclude that the detection of *Rxfp3* mRNA reflects the expression of functional RXFP3 protein by these neurons.

In the MS, VBD, and HDB, the vast majority of *Rxfp3* mRNA-positive neurons co-expressed *vGAT* mRNA. Furthermore, a population of these presumed GABAergic *Rxfp3* mRNA-positive neurons are *PV* mRNA-positive (Wei et al., 2012). Septal PV/GABA neurons are the main source of the GABAergic projections to the hippocampus and specifically target hippocampal interneurons (Freund and Antal, 1988; Freund and Gulyas, 1997). A number of studies have demonstrated that PV/GABA neuron activity is crucial for hippocampal theta rhythm (Borhegyi et al., 2004; Bassant et al., 2005; Simon et al., 2006). The modulation of the GABAergic inter-neuronal inhibition of hippocampal pyramidal neurons has been reported to be a source for hippocampal theta rhythm synchronization (Toth et al., 1997). In addition, septal PV/GABA neurons expressing cyclic nucleotide activated, non-selective cation channels play a role in driving hippocampal theta rhythm (Varga et al., 2008; Hangya et al., 2009). Notably, RXFP3 activation results in inhibition of cellular cAMP synthesis in cell-based assays *in vitro* (Liu et al., 2003; van der Westhuizen et al., 2007, 2010), consistent with a similar interaction *in vivo* (see further discussion below).

In contrast to the strong association with GABAergic neurons, only a small number of cholinergic (*ChAT* mRNA-positive) neurons co-expressed *Rxfp3* mRNA. However, anterograde neural tract-tracing and immunohistochemical studies suggest that cholinergic (ChAT-positive) septal neurons receive a robust innervation from the Rln3 rich NI (Olucha-Bordonau et al., 2012). Thus, the influence of NI neurons on the septal cholinergic system might be mediated by NI neurons that contain GABA only or other peptides, such as cholecystokinin, which is expressed in the NI (Kubota et al., 1983; Olucha-Bordonau et al., 2003) (Ma and Gundlach, unpublished data).

The discovery that *Rxfp3* mRNA is absent from MS cholinergic neurons provides new insights into the nature of the coordinated neural actions that result in the generation and modulation of hippocampal theta rhythm, and since RXFP3 activation often produces neuronal inhibition *in vitro* (Blasiak et al., 2013; Kania et al., 2017), it is possible that pERK activation in MS cholinergic neurons occurs via RXFP3-mediated inhibition of non-PV, GABAergic interneurons (Leranth and Frotscher, 1989). In this regard, optogenetic activation of cholinergic septohippocampal neurons suppressed ripple sharp waves and enhance theta rhythm oscillations (Vandecasteele et al., 2014) and local circuit inhibitory actions on cholinergic neurons are a primary process in the generation of septal rhythmicity (Leão et al., 2015). Furthermore, icv infusion of an RXFP3 agonist (RXFP3-A2; Shabanpoor et al., 2012) resulted in increased phosphorylation of ERK in the MS, mainly in ChAT-immunoreactive neurons (Albert-Gascó et al., 2017). Given the observed absence of *Rxfp3*- and *ChAT* mRNA-positive neurons in the MS in the present study, and the observation that RXFP3 activation routinely induces neuronal inhibition (Kania et al., 2017), there is a possibility that the pERK activation within the cholinergic neurons occurs via a reduction in local circuit inhibition within the MS.

In addition, ∼25% of the *Rxfp3* mRNA-positive neurons in the MS were non-GABAergic, non-cholinergic in nature. Although further studies are required to better identify the phenotype of these neurons, it is presumed that some or many are glutamatergic neurons, since they constitute ∼25% of the total MS neuron population (Colom et al., 2005; Gritti et al., 2006). Glutamatergic neurons provide both local and septohippocampal projections (Manseau et al., 2005; Henderson et al., 2010; Huh et al., 2010) and interestingly, optogenetic activation of MS glutamatergic neurons produces strong theta rhythm synchronization, mainly mediated by local septal circuits (Robinson et al., 2016).

Considerable data suggest a strong link between RXFP3 activation in the MS and modulation of hippocampal theta rhythm. Hippocampal theta rhythm has been traditionally associated with arousal mechanisms which are directly involved in attentional mechanisms of memory (Vinogradova, 1995). The NI, along with other brainstem areas, the hypothalamus and the basal forebrain, promote arousal and fast electroencephalographic (EEG) rhythms (Brown and McKenna, 2015; Korotkova et al., 2018). Moreover, stimulation of the NI promotes arousal and is associated with cortical EEG desynchronization, increased locomotor activity, and head-scanning vigilance behavior during fear recall (Ma et al., 2017). In addition, ipsilateral NI stimulation induces locomotion and rotation at latencies consistent with a role in the modulation of premotor areas like the basal forebrain (Farooq et al., 2016). Furthermore, *Rln3* and *Rxfp3* gene knockout mice display reduced voluntary running wheel activity during the dark, active phase (Smith et al., 2012; Hosken et al., 2015) providing further evidence for a likely role for this signaling system in sustained arousal and related locomotor and exploratory activity.

Indeed, the MS controls exploratory behavior (Köhler and Srebro, 1980; Poucet, 1989; Mamad et al., 2015; Gangadharan et al., 2016). Different forms of memory, including spatial working memory and object recognition can be affected by manipulations of the MS (Givens and Olton, 1994; Fitz et al., 2008; Roland et al., 2014; Okada et al., 2015; Gangadharan et al., 2016). Interestingly, interference with global or septal Rln3/RXFP3 signaling in the rat results in disruption of spatial working memory in the spontaneous alternation test (Ma et al., 2009; Albert-Gascó et al., 2017).

## Conclusion

The strong expression of *Rxfp3* mRNA by GABAergic neurons in the rat MS and adjacent nuclei, is consistent with the central role of these neurons in the control of hippocampal theta rhythm by actions on local septal circuits. In turn, these actions may indirectly influence septal cholinergic neurons/circuits and hippocampal interneurons via septohippocampal projections. Notably, independent studies have revealed a strong Rln3 innervation of the hippocampus and identified *Rxfp3* mRNA expression by hippocampal GABA neurons in the rat (Ma and Gundlach, unpublished data), consistent with direct actions of Rln3/RXFP3 signaling on these circuits. Therefore, further studies of the neurotransmitter and neurochemical phenotype of septal and hippocampal neurons that express *Rxfp3* mRNA and their precise functional roles are warranted in both normal adult rats and mice, and in models of neuropathology and cognitive and psychiatric disorders.

## Author Contributions

HA-G and SM designed and performed the experiments, prepared the figures and drafted the manuscript. FR-B and AMS-P contributed to some experiments, figure preparation and corrections of the draft manuscript. ALG and FEO-B planned the research, supervised the experiments, edited the figures, and prepared the final version of the manuscript.

## Conflict of Interest Statement

The authors declare that the research was conducted in the absence of any commercial or financial relationships that could be construed as a potential conflict of interest.

## Abbreviations

ChAT: choline acetyl transferase
HDB: horizontal diagonal band
LS: lateral septum
LSD: lateral septum dorsal
LSI: lateral septum intermediate
LSV: lateral septum ventral
LV: lateral ventricle
MS: medial septum
NI: nucleus incertus
nNOS: neuronal nitric oxide synthase
PV: parvalbumin
Rln3: relaxin-3
Rxfp3: relaxin-family peptide receptor 3
SFi: septofimbrial nucleus
SFO: subfornical organ
TS: triangular septum
VDB: vertical diagonal band
vGAT (slc32a1): vesicular GABA transporter
